# Version or High Forceps, Which and When?1Read at the thirty-ninth annual meeting of the Medical Association of Central New York, at Syracuse, October 16, 1906,

**Published:** 1907-04

**Authors:** William M. Brown

**Affiliations:** Rochester, N. Y.


### Version or High Forceps, Which and When?[1]

By WILLIAM M. BROWN, M. D., Rochester, N. Y.

IF I were to attempt to give an accurate answer to the above query we would be led into an exhaustive consideration of the whole subject of dystocia and pathological labor, with the indications for and technic of both the high forceps and version operations. I shall not undertake so vast a subject, but the circumstances which attend the need of extraction from the pelvic brim are generally so serious and imperative, and are so often not recognised by the average attendant until both nature and himself have failed to deliver by the ordinary means, by which time the difficulties of an operative delivery are manifolded to the serious menace of both mother and child, and because of the exactions of his other work, the ordinary medical man is illy qualified to execute that extraction to the best interest of the patients who are the subject of his manipulations, that I am led to ask you to bear with me as patiently as possible while I discuss as briefly as I can, what renders operative interference at the superior straight necessary, when to exercise that interference and what that interference shall consist of. I say at the superior straight because a head that is well engaged is a mid-forceps application and is more safely extracted with forceps than by version if, indeed, version is possible at that time.

An essential prerequisite to satisfactory operative aid in labor, is a definitely accurate diagnosis of all the elements which enter into that particular case, which will afford a good working knowledge of the relations which exist between the mother and child and what the possibilities of that relation are. A trained accoucheur is able to estimate these elements in a given case with a fair degree of accuracy.

Now, while the use of the abdominal route for delivery in pathological labor is becoming much more general, we will reserve for some future discussion the question of Cesarean section and today take up only those cases in which it is possible to extract the child through the pelvis. Dystocia may be due to anomalies of the expulsive forces or to an abnormal condition of the generative tract, and Williams describes them under four heads as follows:

1:—Those in which the expulsive forces are subnormal and are not strong enough to overcome the natural resistance offered to the birth of the child by the bony canal and the maternal soft parts.

---

1. Read at the thirty-ninth annual meeting of the Medical Association of Central New York, at Syracuse, October 16, 1906.

2:—Those in which though the expulsive forces may be of normal strength, abnormalities in the structure or character of the birth canal offer a mechanical obstacle to the descent of the presenting part.

3:—Those in which the fetus on account of faulty presentation or excessive development cannot be extruded by the 'vis a a tergo.'

4:—Those cases in which accidental complications such as eclampsia, hemorrhage, etc., lead to various irregularities which interfere with the normal progress of labor.

As before noted, the most essential requisite for satisfactory work in operative obstetrics, and by that I mean not to be satisfied with merely being able to tear a child out and have the mother survive, or perhaps both mother and child, but maimed and mangled to future misery, but satisfactory in that the extraction can be accomplished with a minimum of injury to either mother or child, is a most painstaking examination and study of each individual case. Alas! in the present day of comprehensive practice how prone are we to grasp each case that crosses our path, glad that we have it, but how apt are most of us, in the rush for more, to forget to hold fast to that which we have. Careless study of our cases predisposes to accidents and accidents are fatal to the confidence of our patients. There are few branches of our art where accidents may be so surely anticipated and avoided or their effects minimized, as in the department of obstetrics. With this digression I return to the consideration of the subject of this paper.

When shall we interfere in a labor with the head at the pelvic brim, and shall that interference be a version or a high forceps extraction? Having eliminated those cases of dystocia where an absolute indication for abdominal section is present, there yet remain those cases in which, by reason of a disproportion between the size of the head and the brim of the pelvis, it is difficult or impossible for the uterine muscle to force the head to engagement, or those cases which, by reason of impending or actual danger to either mother or child, require a very rapid emptying of the uterus. Such conditions may be toxemia with profound sepsis or impending or occurred eclampsia, or, perhaps, a placental separation with concealed hemorrhage.

From this we see that there may be two great conditions which require operative procedure with the head at the superior straight; the one requires aid but not necessarily any great haste in the delivery, while the other demands the speediest possible termination of the labor.

It seems to me that the conditions precedent and etiological to the operation give us the key by which we may answer our question, for version, when possible, is the operation of speed although it is often more dangerous to the life of the child; while the high forceps delivery is the one which takes a longer time and does not embrace some of the certain dangers of the version. This is more apparent to us when we recall briefly the details of the two operations.

It is hardly necessary to mention the absolute necessity of perfect surgical cleanliness in the major operations of obstetrical practice as well as in every case of labor, for every labor case is a surgical operation the successful conduct of which is just as dependent upon careful aseptic preparation as any other surgical operation. Therefore, having carefully prepared the patient for the operation, which should include, also, that of the operator to do the work, the next thing is to thoroughly and adequately dilate the canal through which the child has to pass, from the vulva to the internal os. Having decided that the mother cannot or will not be allowed to do the dilatation, then it must be done by the obstetrician. If you have elected to be the force that is to deliver the child, then you are to do the work with your own hands and not with the child's head. Starting, then, at the vulva you proceed to dilate and when you think you have dilated sufficiently then dilate some more, for upon the thoroughness of the dilatation will depend, in a great measure, the success of the delivery.

When the vulva has been sufficiently dilated the vagina should be dilated until the cervix is reached. The hardest part of this division of the operation is the dilatation of the cervix, and sometimes it is almost impossible to accomplish it within the time which circumstances may demand that the labor shall be terminated. In such an instance some more radical procedure will be imperative; but if the child is delivered through the pelvis it is essential that the cervix shall be completely dilated and paralysed. Having accomplished the dilatation in a satisfactory manner the first step in either a version or forceps operation has been accomplished and we may now proceed to do whichever method of delivery under the circumstances has been determined upon. If moments are precious and the danger is more to the mother than to the child, version has probably been chosen, unless the membranes have been ruptured too long, in which case the operator passes his hand into the uterus, as a rule the left hand of the surgeon in left positions of the child and the right hand in right positions.

If the membranes have not been ruptured already, they should not be torn until the hand is well into the cervix, for the presence of a good amount of amniotic fluid renders a version much easier and safer. Passing the hand up in front of the child's face and abdomen a foot is sought. It is not necessary to waste time looking for both feet, but it is generally safer to get the anterior one if possible; but if this takes too much time to determine the foot first grasped may be brought down. It will generally rotate so as to become the anterior one during the extraction. Having brought the foot out at the vulva it is grasped with a sterile towel and steady traction downward and backward is made and, as a rule, the child is delivered as far as the shoulders quite easily, but now the danger point for the child is reached and the thoroughness of the dilatation may decide its fate. If the cervix has not been completely obliterated and paralysed it is prone to shut down as the rest of the uterine muscle retracts, grasping the child by the neck and holding it beyond the time that the child can live unless, by a forceps application and a cervical rupture, you can hasten it. On the other hand, if the dilatation has been adequate, with an assistant making pressure through the abdomen the head may be delivered quite speedily. The operation of turning and extraction should not take more than five to ten minutes.

What is a high forceps operation? As before stated it is not an application to the head in the pelvis. It is an application of forceps to the head before it has engaged and is lying free or wedged at the brim of the pelvis. The same careful preparation and dilatation should precede a forceps application as the version operation. This having been accomplished the patient is ready for the direct placing of the blades of the forceps. For this a blade of sufficient length and fitted with traction rods should be used, as traction in the proper direction cannot be made without them. The head will lie almost transversely at the brim and, because of the normal curve of the bony canal, it cannot be delivered or rather pulled into the pelvis with the forceps applied to the sides of the head.

This is the one condition in which the forceps should not be applied to the sides of the head, but to the brow and mastoid and great care becomes necessary to avoid injury to the child. I have seen an interne deliver a child with an eye gouged from its socket. When the forceps have been properly adjusted traction should be made with the rods in the axis of the pelvic inlet. When the head is well drawn into the pelvis the forceps should be removed and reapplied, this time being adjusted to the sides of the child's head, when rotation is completed and the head delivered in the usual manner. Safe delivery absolutely demands the readjustment of the forceps when the head has been drawn into the pelvis. It was a failure to do this that resulted in the loss of an eye just mentioned.

Now having before our minds the conditions which render an operative delivery necessary and the details of what that operation shall consist of, we are able to take an individual case illumined by this knowledge and estimate with reasonably good judgment what procedure to adopt. A case of eclampsia or placenta prævia should indicate version if possible, while a small pelvis, large head, or uterine inertia would ordinarily mean a high forceps operation.

666 EAST AVENUE.

